# Identification of Novel Gene Targets and Functions of p21-Activated Kinase 1 during DNA Damage by Gene Expression Profiling

**DOI:** 10.1371/journal.pone.0066585

**Published:** 2013-08-12

**Authors:** Mona Motwani, Da-Qiang Li, Anelia Horvath, Rakesh Kumar

**Affiliations:** 1 McCormick Genomic and Proteomics Center, The George Washington University, Washington, District of Columbia, United States of America; 2 Department of Biochemistry and Molecular Medicine, School of Medicine and Health Sciences, The George Washington University, Washington, District of Columbia, United States of America; Peking University Health Science Center, China

## Abstract

P21-activated kinase 1 (PAK1), a serine/threonine protein kinase, modulates many cellular processes by phosphorylating its downstream substrates. In addition to its role in the cytoplasm, PAK1 also affects gene transcription due to its nuclear localization and association with chromatin. It is now recognized that PAK1 kinase activity and its nuclear translocation are rapidly stimulated by ionizing radiation (IR), and that PAK1 activation is a component of the DNA damage response. Owing to the role of PAK1 in the cell survival, its association with the chromatin, and now, stimulation by ionizing radiation, we hypothesize that PAK1 may be contributing to modulation of genes with roles in cellular processes that might be important in the DNA damage response. The purpose of this study was to identify new PAK1 targets in response to ionizing radiation with putative role in the DNA damage response. We examined the effect of IR on the gene expression patterns in the murine embryonic fibroblasts with or without *Pak1* using microarray technology. Differentially expressed transcripts were identified using Gene Spring GX 10.0.2. Pathway, network, functional analyses and gene family classification were carried out using Kyoto Encyclopedia of Genes and Genomes (KEGG), Ingenuity Pathway, Gene Ontology and PANTHER respectively. Selective targets of PAK1 were validated by RT-qPCR. For the first time, we provide a genome-wide analysis of PAK1 and identify its targets with potential roles in the DNA damage response. Gene Ontology analysis identified genes in the IR-stimulated cells that were involved in cell cycle arrest and cell death. Pathway analysis revealed p53 pathway being most influenced by IR responsive, PAK1 targets. Gene family of transcription factors was over represented and gene networks involved in DNA replication, repair and cellular signaling were identified. In brief, this study identifies novel PAK1 dependent IR responsive genes which reveal new aspects of PAK1 biology.

## Introduction

The mammalian PAK family contains six serine/threonine kinases divided into two subgroups, group I (PAK 1–3) and group II (PAK 4–6) on the basis of structural and functional similarities [1]–[3]. Although members of this family share significant homology in the kinase domain , the biological functions of each member are distinct and they are dictated by the variable N-terminal regulatory domain [1], [2].

Among them, PAK1 is the founding and best-characterized member of this family, originally discovered in rat brain as a serine/threonine protein kinase was found to be activated by the P21ras-related proteins Cdc42 and Rac1 [4]. To date, it is clear that a variety of extracellular signals, such as growth factors [5], insulin [6], and lipids [3], can activate PAK1 by promoting its auto-phosphorylation on several sites [1]–[3]. Once activated, PAK1 phosphorylates its downstream substrates, that are responsible for various biological effects of PAK1 kinase in cancer cells [1], [3]. In this context, studies have shown that PAK1 regulates actin cytoskeleton that is crucial for cell morphogenesis, motility, adhesion and cytokinesis by phosphorylating several downstream substrates [3], [7]–[9]. PAK1 also promotes cell survival through direct phosphorylation-induced BAD inactivation [10] and indirectly through several substrates, including NF-κB-inducing kinases [11] dynein light-chain 1 [12] and fork-head transcription factor in response to various stimuli. Furthermore, increased PAK1 expression and activity have been documented in a variety of human cancers, including breast, colon, ovarian, bladder, and brain cancers [1], [3].

In addition to its well-characterized kinase activity, it is increasingly recognized that PAK1 also affects nuclear events, presumably by modulating coactivator/corepressor mediated gene regulation [13]. Earlier studies have demonstrated that PAK1 could be localized in the nuclear compartment and nuclear PAK1 associates with chromatin, suggesting that it might be involved in gene transcription [13]. In support of this notion, nuclear PAK1 has been shown to interact with the phosphofructokinase-muscle isoform (*PFK-M*) and nuclear factor of activated T-cell (*NFAT1*) genes and is involved in regulating their transcription [13]. Furthermore, it has been shown that PAK1 phosphorylates histone H3 on serine 10, a site important in chromosome condensation and transcriptional activation [14]. PAK1 also regulates cyclin D1 transcription by means of a nuclear factor-kappa B (NF-κB)-dependent pathway [15]. In this case, increased expression of PAK1 in breast cancer cells stimulates cyclin D1 promoter activity, increases cyclin D1 mRNA levels, and promotes nuclear accumulation of cyclin D1 [15]. Collectively, these findings suggest that PAK1 could have a role in transcriptional regulation in addition to its putative signaling activity.

More recently, PAK1 signaling has emerged as a component of the DNA damage response as PAK1 status influences the cellular sensitivity to ionizing radiation [16]. However the role of genome-wide PAK1 targets in cells under genotoxic stress such as ionizing radiation remains unknown. Therefore this study was undertaken to identify putative potential PAK1 targets in response to ionizing radiation.

## Results and Discussion

### Strategy to identify potential targets of *Pak1* in IR and non-IR scenarios

**Figure 1:**
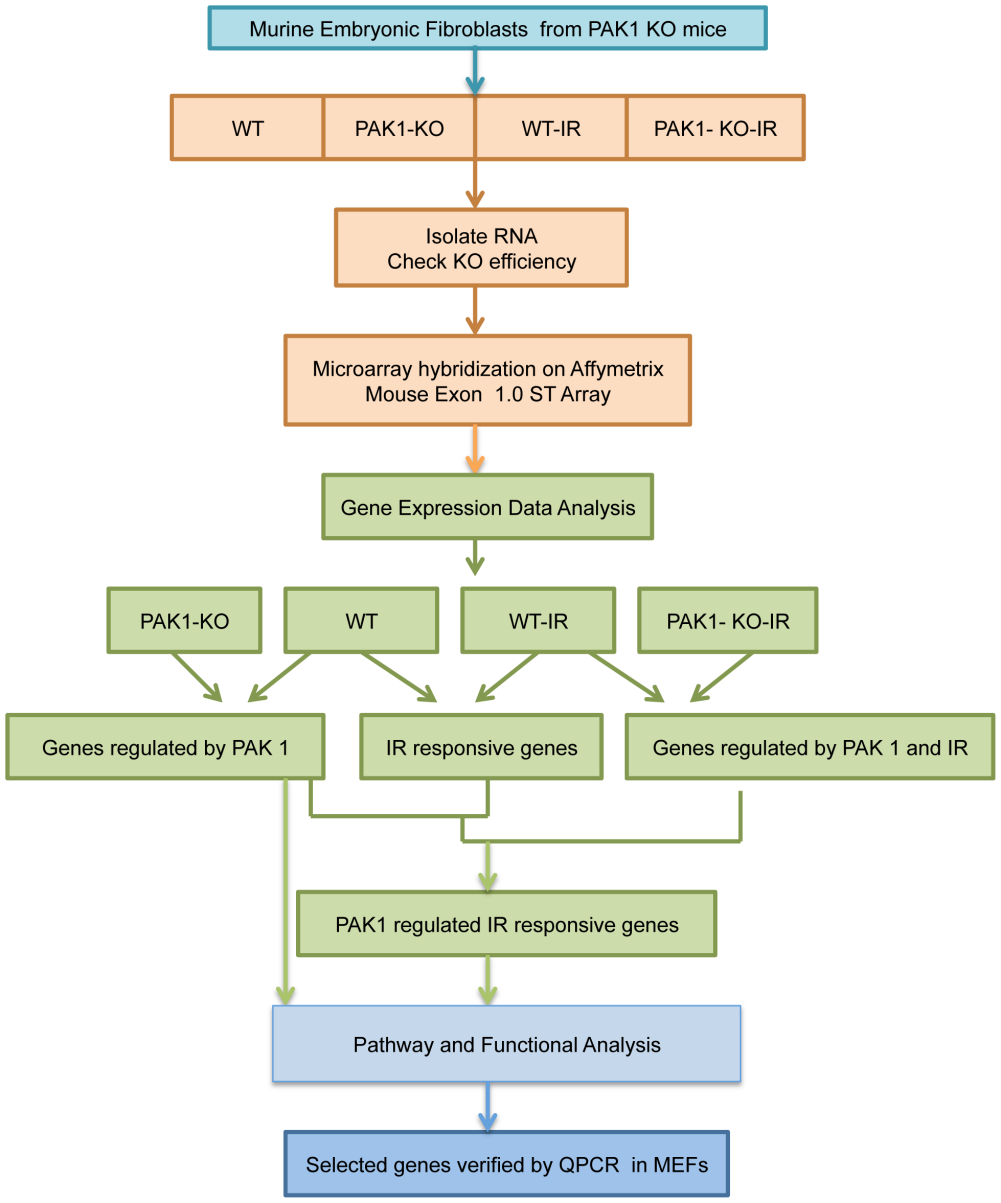
Schematic representing identification of differentially expressed genes by microarray analysis. (**A**) Flowchart representing the global gene expression profiling in the WT and *Pak1* KO and WT and *Pak1* KO IR treated MEFs. RNA was isolated from triplicates of each sample and microarray analysis was performed using the Affymetrix Mouse exon 1.0 chip arrays. The gene expression data was obtained for different samples that were cross compared to identify differentially expressed genes regulated by Pak1 in IR and non-IR scenarios. Functional and pathway analysis was performed to analyze biological significance of differentially regulated genes. Selected genes were verified by qPCR in MEFs.

The overall aim of the study was to identify the genes that are regulated by Pak1 in response to DNA damaging agents such as ionizing radiation (IR). To reveal the role of Pak1 on the gene expression, we have subjected the wild-type (WT) and PAK1 knock-out (KO) murine embryonic fibroblasts (MEFs) to microarray analysis using Affymetrix Mouse Exon 1.0 ST chips. Microarray data normalization and analysis was performed using Gene Spring GX 10.0.2 (Agilent Technologies) to obtain lists of probe sets that were significantly affected by knockout of *Pak1*. To identify the genes regulated by PAK1 in response to IR, the WT and PAK1 KO MEFs were subjected to ionizing radiation dose rate of 3.04 Gy/min at room temperature. We deduced the IR responsive gene list by various cross comparisons between genes lists obtained after analyzing wild-type (WT) and wild-type treated with IR (WT-IR) samples and WT-IR and knock-out treated by IR (KO-IR) samples. This strategy allowed us to identify the genes that were regulated by Pak1 and bonafide Pak1 targets during DNA damage. This potential list of genes was subjected to function, pathway and network analysis by using the Gene Ontology (GO) from Gene Spring, Database for Annotation, Visualization and Integrated Discovery (DAVID) tool and Ingenuity Pathways Analysis (IPA), respectively. Protein Analysis Through Evolutionary Relationships (PANTHER) was used to identify overrepresented and underrepresented gene families in the respective data sets. Reverse Transcription-quantitative PCR (RT-qPCR) was performed to validate the microarray gene regulation of the selected candidate genes in the MEFs. These analyses using both non-IR and IR treated cells helped us identify a set of novel Pak1 targets that are specifically regulated during the DNA damage response. The overall experimental workflow is shown in Figure 1.

### Identification of differentially expressed genes in wild-type and *Pak1*-knockout MEFs

Gene Spring GX 10.0.2 was used to analyze the raw data obtained from microarray hybridization. The initial comparison between the wild type and the *Pak1* knock out cell files was performed using unpaired t-test with a p-value less than 0.05. Benjamini Hochberg false discovery rate (FDR) was applied for the multiple corrections. We have identified 731 Pak1 target genes (table S1) with a fold change ≥±2.0 and with the p-value<0.05. The lists of the top 20 genes that were up-or down-regulated in the Pak1-KO MEFs compared to wild-type MEFs are shown in Table 1 and Table 2, respectively. Highly up-regulated (up to 44-fold change) in the Pak1-KO MEFs are *Pdlim1* gene PDZ - LIM domain 1 and *Sgce* gene (sarcoglycan-epsilon) which encodes the epsilon member of the sarcoglycan family . Both PDLIM1 and SGCE are known to play role in human cancers and cytoskeleton signaling [17] [18] [19] [20]. SGCE is a member of transmembrane proteins, which acts as a link between actin cytoskeleton and the extra cellular matrix and is also implicated in neuronal disorder such as myoclonus-dystonia syndrome . PDLIM1, also known as CLP36, a potential tumor antigen in pancreatic carcinoma, is required for actin stress-fiber formation and focal adhesion in choriocarcinoma which corroborates with functions of PAKs of phosphorylating LIM domain kinase that are regulators of cytoskeleton. [21] Other genes, including receptor-interacting serine-threonine kinase 3 (*Ripk3*), four and a half LIM domains 1 (*Fhl1*), and 1-acylglycerol-3-phosphate O-acyltransferase 2 (*Agpat2*), were also significantly up-regulated in the Pak1-KO cells compared to its wild-type counterparts (Table 1). Interestingly, most of the identified targets show coherence with the putative PAK1 function in cytoskeleton dynamics [1], [3], [22].

**Table 1:** List of the top 20 up-regulated genes in the Pak1 KO MEFs compared to wild-type MEFs.

Refseq	Fold change	Regulation	Gene symbol	Gene description
NM_016861	44.63	up	Pdlim1	PDZ and LIM domain 1 (elfin)
NM_001130188	41.29	up	Sgce	sarcoglycan, epsilon
NM_001164108	28.42	up	Ripk3	receptor-interacting serine-threonine kinase 3
NM_133187	24.49	up	Fam198b	family with sequence similarity 198, member B
NM_001077361	22.38	up	Fhl1	four and a half LIM domains 1
NM_026212	18.84	up	Agpat2	1-acylglycerol-3-phosphate O-acyltransferase 2
NM_011671	18.24	up	Ucp2	uncoupling protein 2 (mitochondrial, proton carrier)
NM_008056	16.92	up	Fzd6	frizzled homolog 6 (Drosophila)
NM_001159518	15.73	up	Igfbp7	insulin-like growth factor binding protein 7
NM_010345	15.22	up	Grb10	growth factor receptor bound protein 10
NM_013657	13.85	up	Sema3c	sema domain, immunoglobulin domain (Ig)
NM_010426	13.55	up	Foxf1a	forkhead box F1a
NM_013560	12.16	up	Hspb1	heat shock protein 1
NM_009152	12.00	up	Sema3a	sema domain, immunoglobulin domain (Ig)
NM_181315	11.69	up	Car5b	carbonic anhydrase 5b, mitochondrial
NM_173006	11.08	up	Pon3	paraoxonase 3
NM_133200	10.97	up	P2ry14	purinergic receptor P2Y, G-protein coupled, 14
NM_008397	10.17	up	Itga6	integrin alpha 6
NM_178395	9.87	up	Zdhhc2	zinc finger, DHHC domain containing 2
NM_007494	8.98	up	Ass1	argininosuccinate synthetase 1

**Table 2:** List of the top 20 down -regulated genes in the Pak1 KO MEFs compared to wild-type MEFs.

Refseq	Fold change	Regulation	Gene symbol	Gene description
NM_025949	17.81	down	Rps6ka6	ribosomal protein S6 kinase polypeptide 6
NM_011443	12.38	down	Sox2	SRY-box containing gene 2
NM_008216	11.23	down	Has2	hyaluronan synthase 2
NM_008343	10.79	down	Igfbp3	insulin-like growth factor binding protein 3
NM_012011	9.78	down	Eif2s3y	eukaryotic translation initiation factor 2
NM_134096	9.39	down	Fam19a5	family with sequence similarity 19
NM_009369	9.25	down	Tgfbi	transforming growth factor, beta induced
NM_012008	8.91	down	Ddx3y	DEAD box polypeptide 3,
NM_007539	8.27	down	Bdkrb1	bradykinin receptor, beta 1
NM_026058	7.74	down	Lass4	LAG1 homolog, ceramide synthase 4
NM_009378	7.36	down	Thbd	thrombomodulin
NM_011581	7.19	down	Thbs2	thrombospondin 2
NM_010357	7.18	down	Gsta4	glutathione S-transferase, alpha 4
NM_009484	7.17	down	Uty	ubiquitously transcribed tetratricopeptide
NM_015744	6.96	down	Enpp2	ectonucleotide pyrophosphatase
NM_010308	6.49	down	Gnao1	guanine nucleotide binding protein, alpha O
NM_080288	6.27	down	Elmo1	engulfment and cell motility 1
NM_008973	6.00	down	Ptn	pleiotrophin
NM_175730	5.45	down	Hoxc5	homeobox C5
NM_001037937	5.30	down	Depdc6	DEP domain containing 6

As shown in Table 2, the ribosomal protein S6 kinase/RSK4 was observed to be 17-fold down-regulated in the Pak1-KO cells compared to the wild-type controls. RSK4, an intracellular serine/threonine kinase, belongs to p90 ribosomal S6 kinase (RSK) family of related kinases that phosphorylate numerous cytosolic and nuclear targets and act as downstream effectors of the extracellular signal-regulated kinase/mitogen-activated protein kinase signaling cascade [23]. RSK4 is commonly deleted in patients with complex X-linked mental retardation, suggesting that RSK4 plays a role in normal neuronal development [24]. In addition, RSK4 has been reported to be deleted or down-regulated in many human cancers such as colon [16], [25] and breast cancers [26] [27]. the RSK family of proteins, RSK1 and RSK2 shown to phosphorylate filamin A cytoskeletal protein that crosslinks actin filaments and is essential for mammalian cell motility [28]. Other most significantly down-regulated genes due to Pak1 knockout include insulin-like SRY-box containing gene 2 (*Sox2*), a pluripotent gene and an important transcription factor found overexpressed in various cancers such as colon, lung and breast. It is known to contribute to tumor initiation and propagation in mammary cells as well as donwregulation of this gene suppresses growth and metastasis in lung cancer [29] [30]. It is interesting to note that there are several known functional avenues including neuronal development, cytoskeletal, cell motility signaling, and oncogenic signaling are influenced by Pak1. Our data indicates possible unexplored interplay between such substrates and Pak1 which may be through genomic regulation. Other genes include hyaluronan synthase 2 (*Has2*), eukaryotic translation initiation factor 2, subunit 3, structural gene Y-linked (*Eif2s3y*) and insulin-like growth factor binding protein 3(*Igfbp*) (Table 2). Collectively, these analyses provide a genome-wide view of a set of new Pak1 targets with roles in various biological processes.

### Genes regulated by *Pak1* in response to DNA Damage stimuli

To achieve our aim of identifying the Pak1 regulated genes under ionizing radiation, we performed statistical tests between the wild type cells treated with IR (WT-IR) and *Pak1*-KO cells treated with IR (KO-IR) that used a p-value less than 0.05, fold change ≥±2.0 and the Benjamini Hochberg false discovery rate (FDR) correction parameter to eliminate false positives. This comparison yielded a list of genes that might be differentially regulated due to three main reasons: a) mere knockout of the gene b) effect of IR alone or c) both knockout and IR. The genes that are differentially regulated due to both knock out and IR are likely to be involved in DNA damage response in a *Pak1*-dependent manner. To obtain this set of genes, we eliminated the ‘only IR’ effected genes by comparing the WT and WT-IR samples from the list of WT-IR vs KO-IR. Next we eliminated all the differentially expressed genes that were present due to ‘only KO’ of *Pak1*. At this stage of filtering, we compared the fold changes of the genes between WT vs KO and the WT-IR vs KO-IR. If fold change was same in both cases we eliminated those genes by concluding that the gene is affected by ‘Only KO’ but not IR. One can argue that the up or down regulation of the gene caused due to the KO of *Pak1* in MEFs might be exactly compensated with the effect IR so that in the end there is a null fold change difference. Addressing this point is almost impossible using microarray technology, hence those genes were not taken into consideration and assumption has been made that such genes, if any, might be very few in number.

Therefore, by filtering, we obtained the final list of target genes that might be involved in DNA damage response mechanisms as well as affected by absence of *Pak1* and hence were “bonafide”. A total of 199 genes passed these selection criteria and achieved statistical significance (p-value<0.05 and fold change ≥2.0) (table S2). Top 20 up-regulated and down- regulated *Pak1* influenced genes after ionization radiation treatment are shown in Table 3 and Table 4 respectively. Interestingly, the highly up regulated target genes cyclin dependent kinase inhibitor 1 A (*Cdkn1a*), transformation related protein 53 inducible nuclear protein 1 (*Trp53inp1*), B cell translocation gene 2 (*Btg2*), SET domain containing (lysine methyltransferase) 8, appeared to be clusters of genes involved in cell cycle arrest. The family of genes such as Ectodysplasin A2 receptor, adrenergic receptor, beta 2 pleckstrin homology-like domain, family A, member 3, p53-induced protein (sestrin), G two S phase expressed protein 1 and DNA-damage-inducible transcript 4 have been reported to be involved in P53 signalling in DNA damage. For instance, ectodysplasin A2 receptor, a signaling molecule belonging to the tumor necrosis factor family, required for normal development of several ectodermally derived organs in humans and mice has been shown to be a direct P53 target in colorectal cancer [31]. Another target gene, *PHLDA3*, an established p53 responsive gene that encodes a PH domain-only protein is shown to influence p53-dependent apoptosis through Akt and subsequently established to be a central player in tumor suppression. [32]. Finally, *DDIT4* (DNA damage-inducible transcript 4), a P53 transcriptional target stress-response gene that negatively regulates the mTOR pathway is also upregualted by PAK1 and IR treatment. The mTOR (mammalian target of rapamycin) protein kinase is the central node in nutrient and growth factor signaling, and activation of p53 inhibits mTOR activity, tumor suppression processes such as autophagy and regulates its downstream targets. [33]. In summary, PAK1 appears to be a critical player that is necessary for the genomic regulation of several genes that are involved in p53 mediated DNA damage signaling. Of note, the genomic regulatory functions of PAK1 are at an early stage of understanding and our findings represent a wealthy resource to gain a deeper insight into PAK1 biology.

**Table 3:** List of the top 20 IR responsive genes upregulated by genes in the IR treated Pak1 KO MEFs compared to its IR treated wild-type MEFs.

Refseq	Fold change	Regulation	Gene symbol	Gene description
NM_007669	26.87	up	Cdkn1a	cyclin-dependent kinase inhibitor 1A
NM_021897	23.48	up	Trp53inp1	transformation related protein 53 inducible nuclear protein 1
NM_007570	16.05	up	Btg2	B-cell translocation gene 2, anti-proliferative
NM_009855	15.69	up	Setd8	SET domain containing 8
NM_007494	14.41	up	Ass1	argininosuccinate synthetase 1
NM_030143	14.33	up	Ddit4l	DNA-damage-inducible transcript 4-like
NM_021274	11.79	up	Cxcl10	chemokine (C-X-C motif) ligand 10
NM_021451	9.43	up	Pmaip1	phorbol-12-myristate-13-acetate-induced protein 1
NM_010786	6.05	up	Mdm2	transformed mouse 3T3 cell double minute 2
NM_013473	5.97	up	Anxa8	annexin A8
NM_020275	5.68	up	Tnfrsf10b	tumor necrosis factor receptor superfamily, member 10b
NM_001161432	5.35	up	Eda2r	ectodysplasin A2 receptor
NM_008651	4.48	up	Mybl1	myeloblastosis oncogene-like 1
NM_007420	4.29	up	Adrb2	adrenergic receptor, beta 2
NM_013750	3.88	up	Phlda3	pleckstrin homology-like domain, family A, member 3
NM_144907	3.81	up	Sesn2	sestrin 2
NM_008331	3.72	up	Ifit1	interferon-induced protein with tetratricopeptide repeats 1
NM_024223	3.59	up	Crip2	cysteine rich protein 2
NM_013882	3.52	up	Gtse1	G two S phase expressed protein 1
NM_013642	3.50	up	Dusp1	dual specificity phosphatase 1

**Table 4:** List of the top 20 IR responsive genes down regulated by genes in the IR treated Pak1 KO MEFs compared to its IR treated wild-type MEFs.

Refseq	Fold change	Regulation	Gene symbol	Gene description
NM_007539	6.32	down	Bdkrb1	bradykinin receptor, beta 1
NM_028133	3.35	down	Egln3	EGL nine homolog 3 (C. elegans)
NM_021532	3.22	down	Dact1	dapper homolog 1
NM_008970	2.50	down	Pthlh	parathyroid hormone-like peptide
NM_138747	2.42	down	Nop2	NOP2 nucleolar protein homolog (yeast)
NM_010499	2.39	down	Ier2	immediate early response 2
NM_009227	2.38	down	Snrpe	small nuclear ribonucleoprotein E
NM_001011721	2.35	down	Olfr102	olfactory receptor 102
NM_001161845	2.30	down	Sgk1	serum/glucocorticoid regulated kinase 1
NM_008492	2.30	down	Ldhb	lactate dehydrogenase B
NM_019444	2.29	down	Ramp2	receptor (calcitonin) activity modifying protein 2
NM_021878	2.25	down	Jarid2	jumonji, AT rich interactive domain 2
NM_173011	2.24	down	Idh2	isocitrate dehydrogenase 2
NM_007514	2.22	down	Slc7a2	solute carrier family 7, member 2
NM_001012477	2.19	down	Cxcl12	chemokine (C-X-C motif) ligand 12
NM_010898	2.18	down	Nf2	neurofibromatosis 2
NM_178891	2.18	down	Prmt6	protein arginine N-methyltransferase 6
NM_009930	2.17	down	Col3a1	collagen, type III, alpha 1
NM_025392	2.158529	down	Bccip	BRCA2 and CDKN1A interacting protein
NM_029936	2.1434464	down	Ddx10	DEAD (Asp-Glu-Ala-Asp) box polypeptide 10

### Hierarchical clustering analysis of Pak1 on gene expression with and without IR treatment

**Figure 2:**
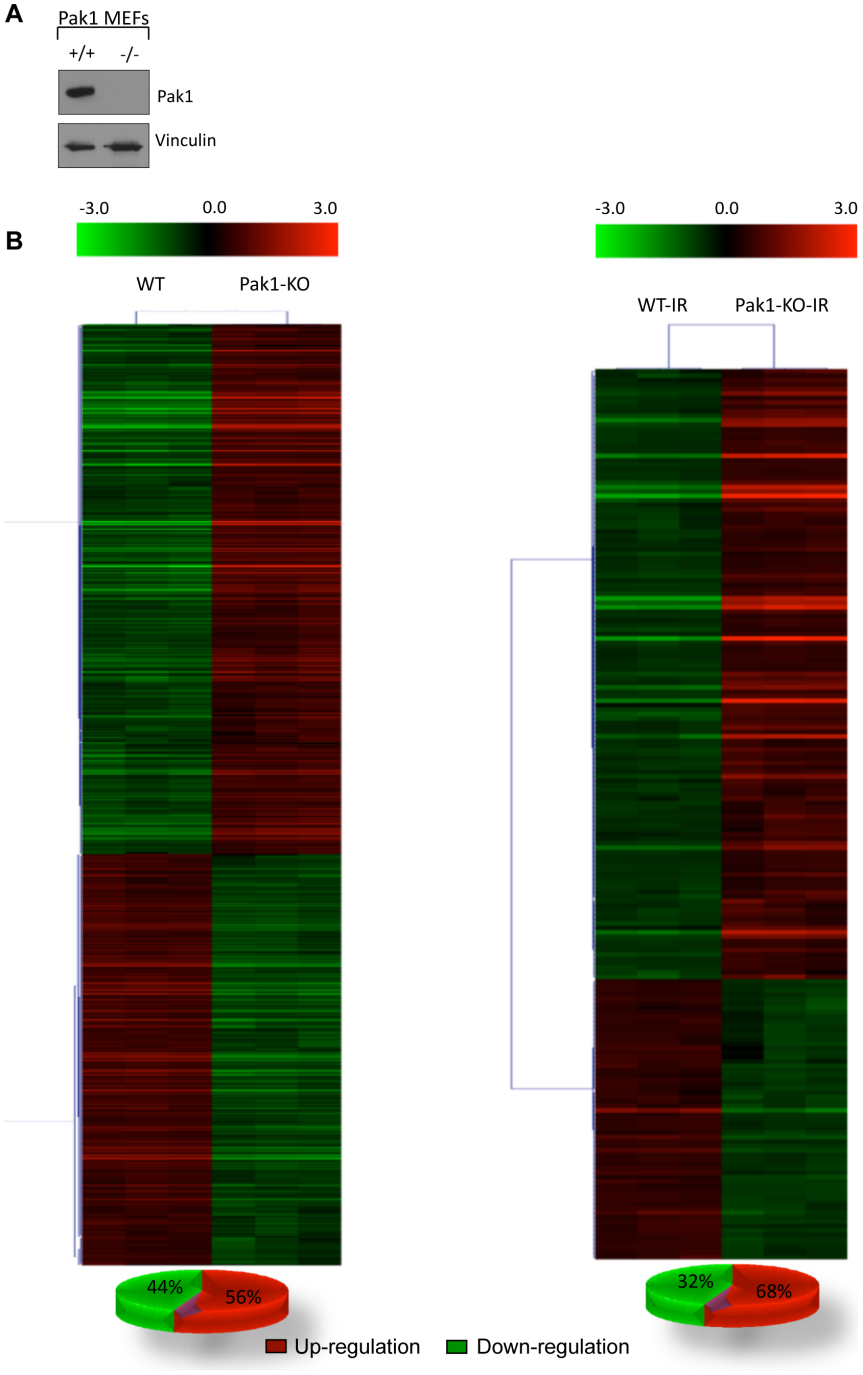
Hierarchical clustering of differentially regulated genes. (A) Western blot analysis of Pak1 in the WT and Pak1 KO MEFs. The same membrane was probed with anti-vinculin as loading control. (B) Heat map shows the hierarchical clustering of the differentially expression genes generated by MultiExperiment Viewer version 4.4. The log2 expression values of the genes are represented on the color scale from green indicating down-regulation to red indicating an up-regulation, interpolated over black region representing no significant change in expression of the genes when *Pak1* is knocked out. The differential expression analysis was performed with *P*<0.05 using Benjamini Hochberg method and fold change ≥2.0.

PAK1 protein level in the WT and KO MEFs were checked using western blotting and is shown in Figure 2A. We performed the hierarchical clustering analysis with the genes that were differentially regulated between the WT vs. Pak1-KO and that were Pak1 regulated, IR responsive genes. The normalized log2 ratio values of the differentially regulated genes in each comparison were used to obtain the heat maps (Figure 2B). The gene leaf nodes were optimized in the heat maps representing the differential regulation of the genes within the samples. Coloring scale of the heat map shows red as the up regulated, green as down regulated and black as insignificant differential expression. We found that 56% of the differentially regulated genes were up-regulated in WT vs. Pak1-KO whereas 44% were down regulated. Following the IR treatment 68% were found to be up regulated and 32% down regulated in the KO samples as compared to the wild type.

### Gene ontology (GO) analysis of *Pak1* knockout with and without IR treatment

**Figure 3:**
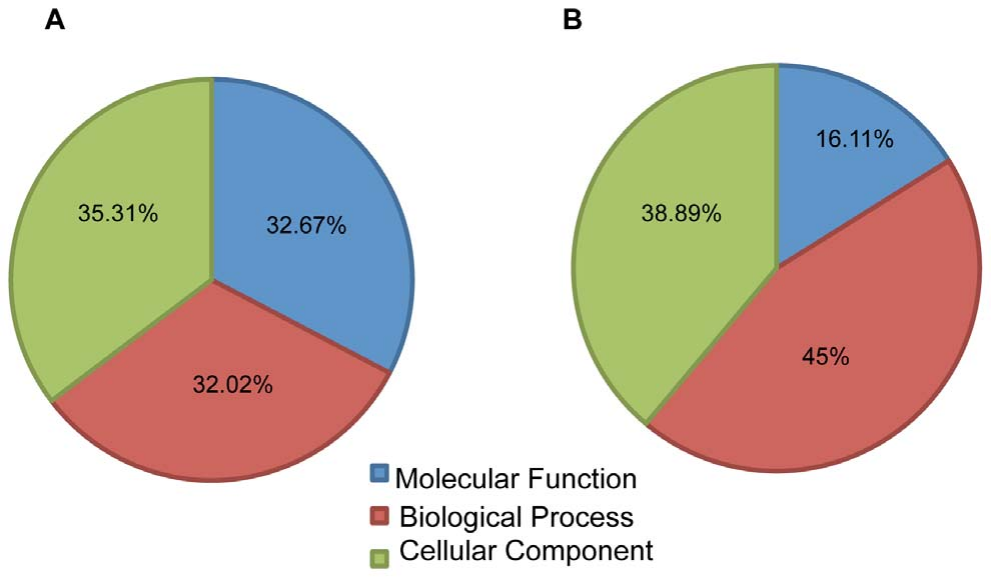
Gene Ontology analysis. The figure represents the pie chart view of the GO analysis performed by Genespring GX 10.0.2. The percentages represent statistically significant (p<0.1) genes that match each GO term. The pie chart sections represent GO terms biological processes, cellular components and molecular functions. The GO analysis gives a broad overview of the biological significance of the differentially regulated genes. Figure (A) represents differentially regulated genes by PAK1 whereas (B) represents differentially expressed genes regulated by Pak1 that were responsive to IR.

**Figure 4:**
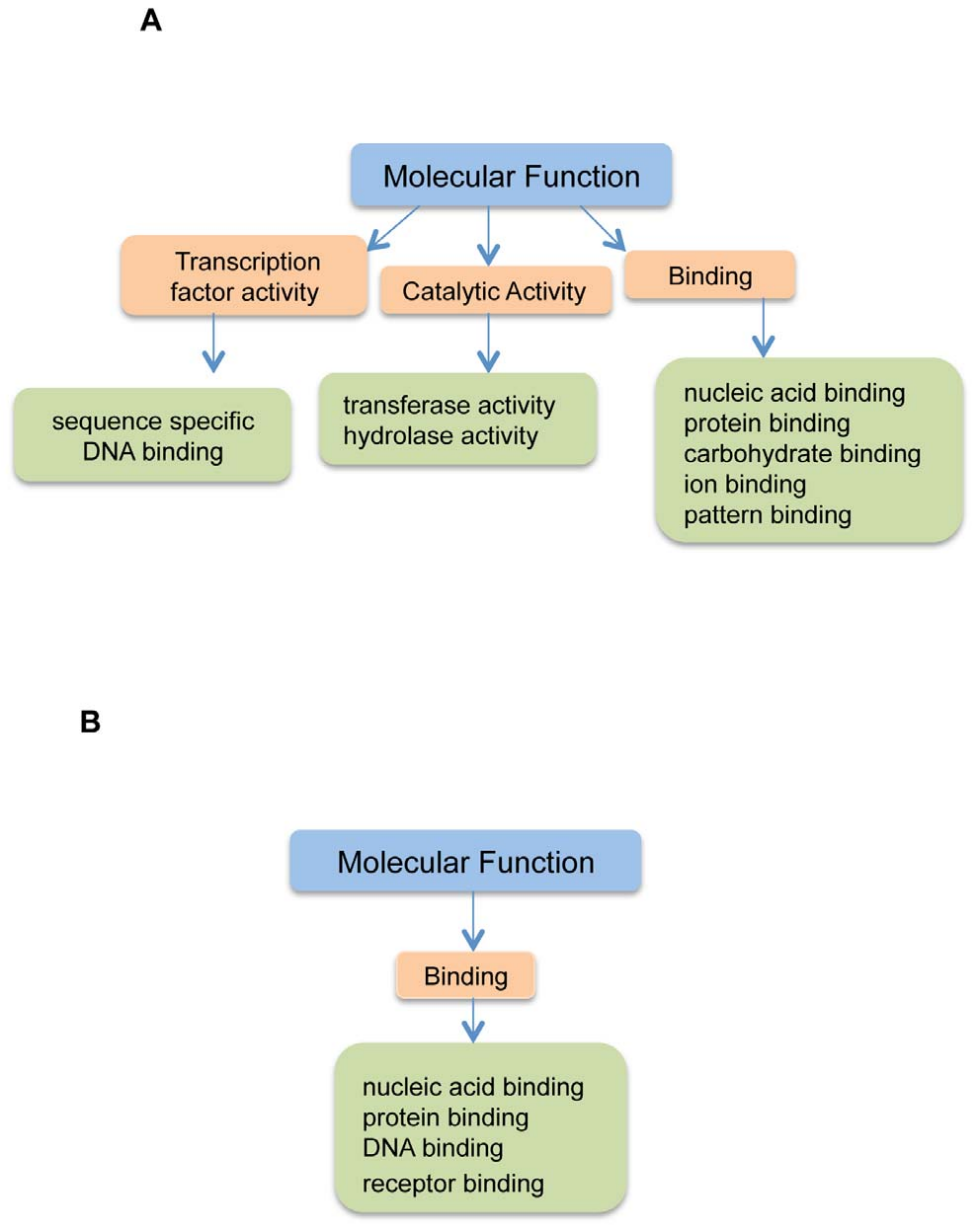
Gene Ontology Tree for Molecular Functions. The Gene Ontology analysis follows a hierarchical way of assigning GO terms to genes. A GO tree is an expansion to lower level terms that allows us to know specific functional categories. Figure (A) represents lower level classifications of molecular functions by PAK1 regulated genes. (B) Shows the same for PAK1 regulated DNA damage responsive genes. All the categories follow statistical significant value of p<0.01. Pak1 regulated genes are involved in molecular functions such as “Transcription factor activity”, “catalytic activity” and “binding” where as Pak1 regulated IR responsive genes were only involved in “binding”. All the categories are statistically significant with P- value<0.01.

**Figure 5:**
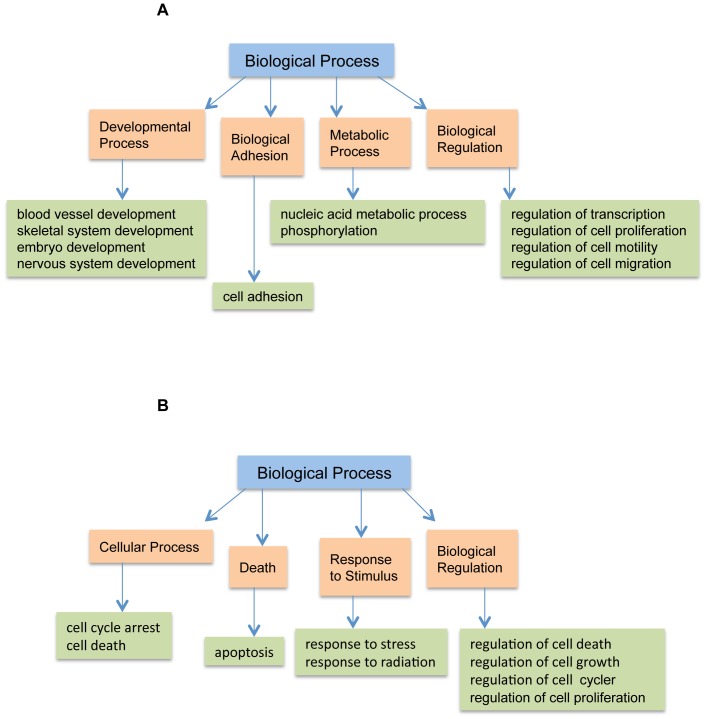
Gene Ontology Tree for Biological Process. A Hierarchical representation of GO terms for biological processes. Panel (A) Represents lower level classifications of biological processes by PAK1 regulated genes which includes developmental processes, cellular adhesion, and regulation of cell motility and cell proliferation. Panel (B) Shows lower level classifications of biological processes for PAK1 regulated DNA damage responsive genes. These genes are mostly involved in cell cycle arrest, cell death, response to stress and apoptosis. All the categories are statistically significant with P- value<0.01.

To understand the functions of these differentially expressed probe sets, we performed GO analysis using Gene Spring GX 10.0.2. on all the sets of genes that were regulated by *Pak*1 and *Pak1*-regulated DNA damage responsive genes with a p-value cutoff set to 0.1. The results showed that 32.02% of these genes matched the GO term “Biological Processes”, 32.67% had “Molecular Functions”, and 35.31% matched “Cellular Component” (Figure 3A). When we analyzed the PAK1 dependent IR regulated genes, a contrasting 16.11% were involved in “Molecular Functions”, 45% in “Biological Processes” and 38.89% matched “Cellular Component” (Figure 3B). To analyze the functions we further expanded the Gene Ontology terms to study biological processes and molecular functions and studied significant processes based their p-values. PAK1 regulated genes were involved in molecular functions such as “catalytic activity” “binding” and “transcription factor activity”. Catalytic activity mainly comprised of hydrolase and transferase activity representing large number of enzymes that were affected by loss of PAK1. Transcription factor activity constituted sequence specific DNA binding genes. This analysis revealed a specific set of genes that were regulated by PAK1 and were crucial for transcription machinery (Figure 4A). Contrasting to this scenario PAK1 dependent IR responsive genes were significantly involved in “binding” which is a broad category that includes all different kinds of binding such as protein binding, nucleic acid binding and receptor binding (Figure 4B). To understand what processes were being affected by the loss of PAK1 kinase we expanded the GO term “Biological Process” and found that PAK 1 regulated genes and PAK1 regulated IR responsive genes regulate distinct processes. PAK1 dependent genes were involved in processes such as “biological adhesion”, “metabolic processes” such as nucleic acid metabolism and phosphorylation, “developmental processes” such as blood vessel development, skeletal system development, embryonic and nervous development, and many “regulatory processes” like regulation of transcription, cell proliferation, cell motility and migration (Figure 5A). The IR set of genes represented very specific processes characteristic of IR scenario such as “cellular process” involving cell cycle arrest, cell death “death” mainly apoptosis, “response to stimulus” like stress and radiation “biological regulation” like regulation of cell death, cell growth, cell cycle and cellular proliferation. (Figure 5B). The Gene Ontology analysis provided an overview of various functions and biological processes in which these genes were involved. Pak1 regulated IR responsive genes were mainly involved in various DNA damage responsive events like cell cycle arrest and apoptosis whereas Pak1 1 KO genes displayed functions in developmental processes. The details of overall GO terms involved are shown in table S3 and S4 for the non-IR and IR scenarios.

### Comparative analysis of pathways and networks in non-IR and IR stimulated scenarios

**Figure 6:**
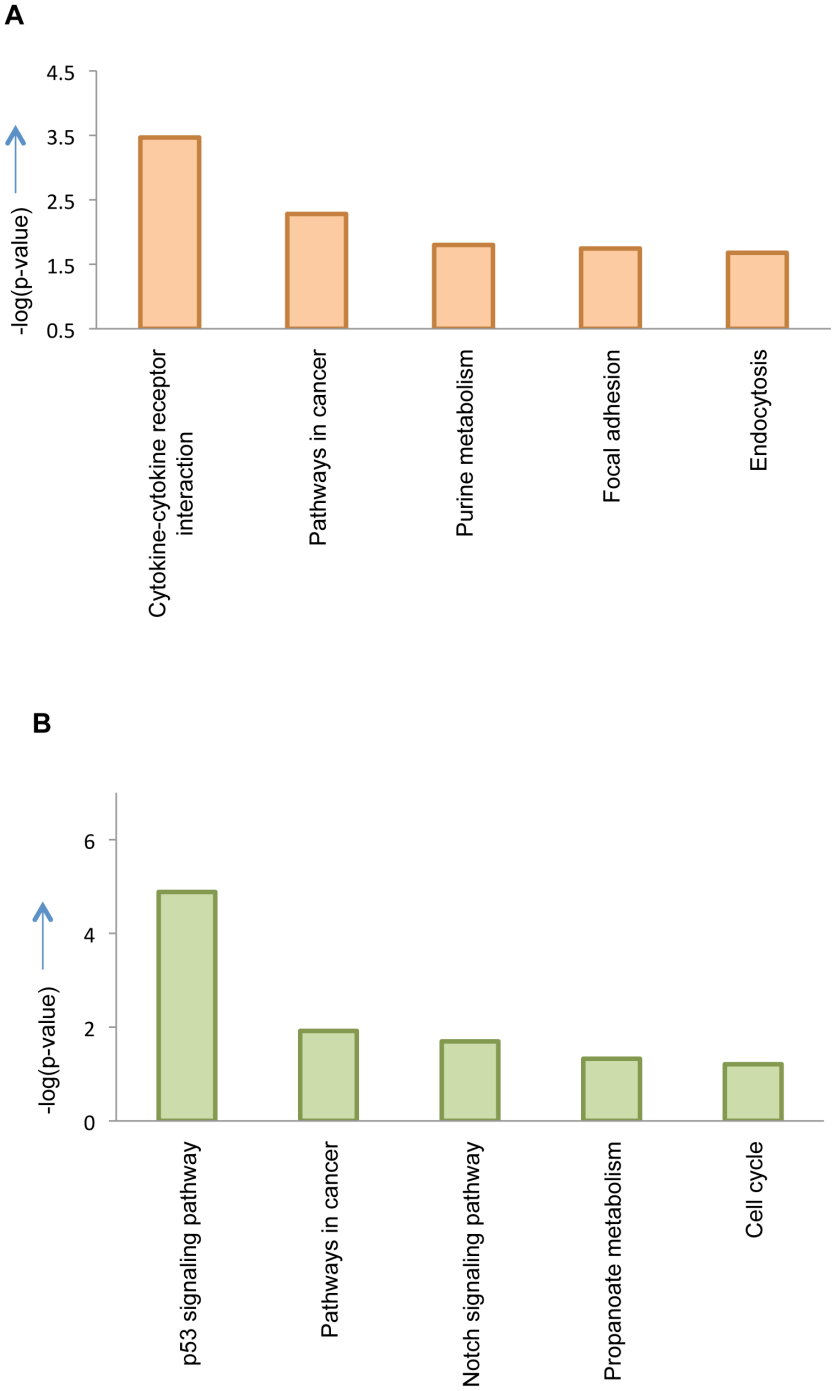
Top five KEGG pathways. DAVID tool was used to analyze differentially regulated genes to identify top pathways that were influenced by these subset of genes. Panel (A) shows the graph representing the top pathways for genes being regulated by PAK1 whereas panel (B) represents pathways for genes regulated by PAK1 in response to DNA damage. The analysis utilized p value<0.1 and Benzamini Hochberg, Bonferroni and FDR corrections were applied for minimize the number of false positives.

To understand if the gene sets were involved in biological pathways we used DAVID 6.7 tool to analyze various KEGG pathways, enriched with the sets of upregulated and down regulated genes. Most of the genes that had altered expression due to loss of Pak1 belong to pathways in cancer cytokine-cytokine receptor interaction, purine metabolism, focal adhesion and endocytosis. As defined by KEGG database ‘Cytokine-cytokine receptor interaction pathway constitutes of cytokines which are crucial intercellular regulators during inflammatory host defenses, cell growth, differentiation, cell death, angiogenesis, and homeostasis’. PAK1 is known to control MAP kinases like JNK and p38 signaling kinases which respond to stress stimuli and regulate cellular processes. [34]. Also the modulation of NF-kB signaling by Pak1 has been well documented. [11]. Cytoskeleton dynamics and formation of stress fibers that are mediated by PAK1 play an important role in cell matrix interactions during an inflammatory event. All these findings support a potential involvement of Pak1 in cytokine-cytokine receptor interaction which has not been explored. Also, Pak1-KO influenced many new targets such as chemokines, platelet derived growth factors, transforming growth factors and tumor necrosis factors that are components of this pathway and haven't been studied with respect to Pak1. Pathways like cancer and focal adhesion pathways represent known functions of Pak1. In contrast, Pak1 regulated IR responsive genes were involved in pathways such as p53 signaling, cancer, proponoate metabolism and cell cycle. Although the p53 dependent functions of Pak1 have been investigated before [35] here Pak1 appears to regulate several target genes that are involved in p53 signaling, which have long been established to be central to DNA damage.. The P53 protein plays a pivotal role in DNA damage response and tumor suppression and depending on the stress signal various signaling cascades are activated. Important genes such as *Mdm2*(6 fold upregulated in knock out samples) invloved in cell cycle and p53 feedback loop, *Apaf1*(2.6 fold upregulated) and *Noxa* which are involved in apoptosis, *Sestrins(*3.8 fold upregulated) responsible for DNA damage repair were shown be altered due to loss of Pak1. The top 5 pathways for the non-IR and IR set are shown in Figure 6a and 6b respectively and the remaining significant pathways are shown in Tables 5 and 6. . The p-value used to compute the KEGG pathways was equal to 0.1, Bonferroni and Benjaminicorrections were applied for the analysis. (tables S5 and S6).

**Table 5:** The top KEGG pathways by DAVID tool in non-IR scenario.

Term	Count	% of genes	P-Value
Cytokine-cytokine receptor interaction	22	3.09	3.41E-04
Pathways in cancer	23	3.23	0.00523118
Purine metabolism	13	1.82	0.0158467
Focal adhesion	15	2.10	0.01796474
Endocytosis	15	2.10	0.02099121
Axon guidance	11	1.54	0.02672229
Toll-like receptor signaling pathway	9	1.26	0.03380387
Metabolism of xenobiotics by cytochrome P450	7	0.98	0.03836115
Chemokine signaling pathway	13	1.82	0.04350035
p53 signaling pathway	7	0.98	0.04613989
Cytosolic DNA-sensing pathway	6	0.84	0.05656196
Drug metabolism	7	0.98	0.06445811
Bladder cancer	5	0.70	0.0737393
NOD-like receptor signaling pathway	6	0.84	0.08537709

**Table 6:** The top KEGG pathways by DAVID tool in IR scenario.

Term	Count	% of genes	P-Value
p53 signaling pathway	8	4.10	1.31E-05
Pathways in cancer	10	5.12	0.012073
Bladder cancer	4	2.05	0.012626
Notch signaling pathway	4	2.05	0.020181
Glioma	4	2.05	0.038248
Propanoate metabolism	3	1.53	0.047369
Melanoma	4	2.05	0.049558
Chronic myeloid leukemia	4	2.05	0.058529
Cell cycle	5	2.56	0.061736
Cytokine-cytokine receptor interaction	7	3.58	0.064104
ErbB signaling pathway	4	2.05	0.080734
Apoptosis	4	2.05	0.080734
Prostate cancer	4	2.05	0.087345
MAPK signaling pathway	7	3.58	0.087658

These findings were reconfirmed using Ingenuity Pathway Analysis (IPA) (release Winter 2012) to generate a network which aggregates the genes from our data set based on known relationships, functions and diseases. The top network of Pak1 regulated genes involved “Cellular Movement, Cancer, Tissue Development”, which is in coherence with GO terms such as “regulation of cell motility”. Similarly, tissue development incorporates GO term “developmental system”. Thus, by applying two different algorithms, our analysis highlighted related pathways and functions. The network analysis brings out the major theme in Pak1 regulated genes. Similarly, the top network in Pak1 dependent IR responsive genes was the “Cellular Growth and Proliferation, DNA Replication, Recombination, and Repair, Cell Signaling” which establishes the main theme found in these sets of genes which is in agreement with the GO term functional analysis and KEGG pathways. The top 5 networks by IPA in both non-IR and IR scenarios are provided in table S7.

### Gene family classification

**Figure 7:**
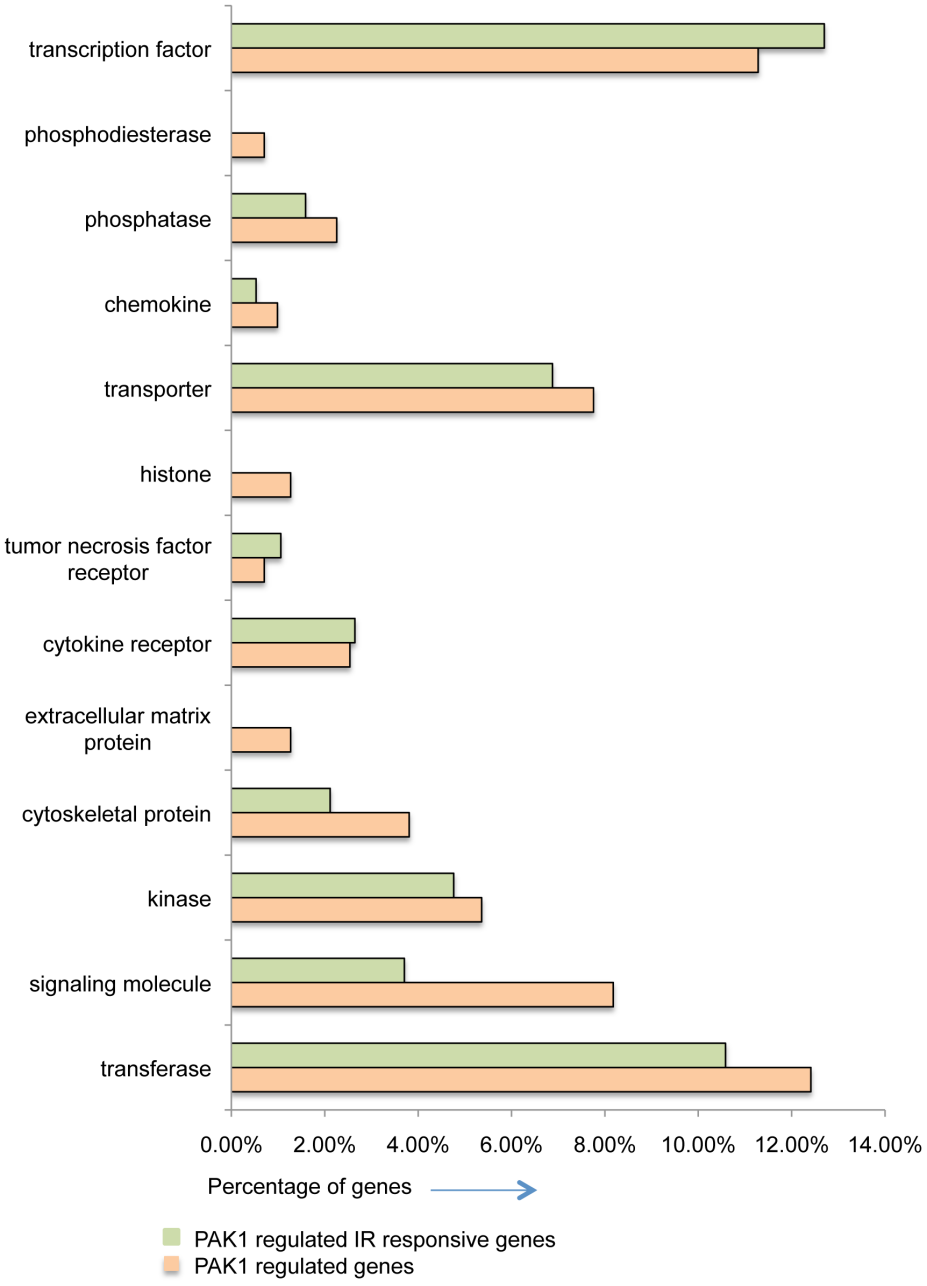
Gene family classification from PANTHER. This figure represents various gene families that are over or under represented for both IR and non-IR scenarios. The over and underrepresented families are computed by comparing the gene families in our list to a reference genome which in this case was *Mus Musculus*. Binomial distribution is applied to obtain p-values and p values<0.05 were considered. Percentage of genes for each gene family that were differentially represented are shown in the figure. Transcription factors are the most over-represented gene family in both IR and non-IR conditions.

Having established the differentiated pathways and functions of Pak1 in IR and non-IR scenarios, we then explored the possibilities of over or under represented gene families in these sets of lists using PANTHER tool (version 7.0). PANTHER protein class tool compares the set of altered genes to the reference genome, in this case Mus Musculus, and computes if our data set is enriched with categories of gene or protein families(table S8). We chose the top gene families (Figure 7) based on their p-values from both the IR and non-IR sets and observed that three classes of gene families which were transcription factors, transferases and transporters were over represented for both the sets, whereas gene families like the histones and extracellular matrix genes were specific to only Pak1 regulated genes and were underrepresented in IR scenario. Tumor necrosis receptor family and transcription factors were over represented in IR scenarios. These gene families were also implicated in functions that were explored using GO analysis. For example, transferases were an important component of molecular functions like DNA binding and catalytic activity, many transporters involved in enzyme activity and signaling molecules, were implicated in biological processes like cellular regulation. We can see that transcription factors are the major players of genomic regulation through Pak1 in both IR and non-IR scenarios. Transcriptional targets of Pak1 have been extensively studied with respect to cytoskeleton remodeling like the estrogen receptor α (ERα), Forkhead transcription factor (Fkhr), SHARP and EMT transitions [36] but here for the first time we explore the transcriptional targets of Pak1 in many different cellular processes and in response to DNA damage which can either be directly or indirectly influenced by PAK1. To understand the transcriptional regulation of Pak1 we further analyzed if there were any distinct transcription factors governing the characteristic features of Pak1 targets in IR and non-IR scenarios.

### Transcription Factors analysis by IPA

**Figure 8:**
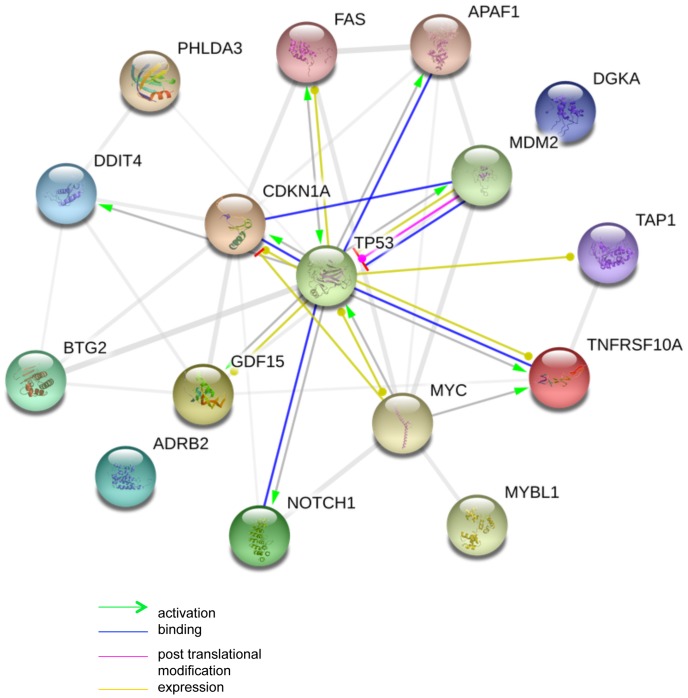
Network of P53 and its targets regulated by PAK1 using STRING database. In the figure transcription factor P53 and its targets are represented. Brown lines represent expression; blue lines represent binding, green lines represent activation of the target molecules whereas pink lines represent post translational modifications mediated by P53. MDM2, Ddit4, PHLDA3, CDKN1A are few of the P53 targets that are also influenced by *Pak1* knock out.

Since we found that transcription factors were over represented in the gene families we performed the transcription factor analysis from IPA (release Winter 2012) to find the top transcription factors regulating majority of targets in both IR and non-IR scenarios. Distinct set of transcription factors were up- and down- regulated in the two scenarios. The non-IR scenario consisted of transcription factors as CEBPA, VDR, SPDEF, MLL2, and NR3C1, whereas the IR scenario comprised of TP53, ESR2, NFkB (complex), TP63, and JUN. CEBPA, CCAAT/enhancer-binding protein alpha transcription factor is deregulated in acute myeloid leukemia which results in differentiation and increased proliferation of cells. The CEBPA is thought to be deregulated post translationally, by RNA interference or through transcriptional silencing [37]. CEBPA is predicted to regulate the expression of these upregulated or down regulated genes directly or indirectly. Few targets of this transcription factor are BTG2, CSF1R, DYNLT3, EPHX1, MT2A, and VDR. We observe that VDR being a transcription factor is one of the targets of CEBPA. This kind of analysis provides insights to common targets of the transcription factors as well as the regulatory interplay among the transcription factors that might be occurring due to loss of PAK1. In the IR scenario P53 is one of the major transcriptional regulators. A network of P53 and its targets predicted from our gene list is presented at Figure 8. CDKN1A and MDM2 known to bind P53 [38] [39], the other genes that are predicted to be regulated by P53 are DGKA, ADRB2 but have not been explored till date. Such analysis and visualization can help us infer that if there is a genomic regulation between PAK1 and TP53 then all these associated molecules will be affected by this interplay and will have an effect on the downstream signaling pathways. The top 5 transcriptional regulators for each scenario are present in Tables 7 and 8 and the entire list is available in Tables S9 and S10.

**Table 7:** Top transcription factors by Ingenuity Pathway Analysis in non-IR scenario.

Transcription Regulator	p-value	Target molecules in dataset
**CEBPA**	2.11E-06	BTG2,CSF1R,DYNLT3,EPHX1,IFI27,MT2A,NFIL3,PFN2,PLOD2,RGS2 ,SERPINF1,SPINT2,SPP1 ,THBD,TUBB2A,VDR
**VDR**	5.39E-06	CDKN1A,IGFBP3,PLCG1,THBD,UCP2
**SPDEF**	5.78E-06	BIRC3,CD34,CDH11,COL4A2,COL4A6,COL5A2,CTGF,ITGA6,PLAU,SMAD1,TGFB1
**MLL2**	1.21E-05	CRIP1,CRIP2,ENO3,FHL1,GPR56,GPR64,NPR3
**NR3C1**	2.58E-05	AKTIP,BIRC3,CASP4,CDKN1A,CORO2A,CTNNBL1,CUL4A,GEM,IFIH1,IL6,ING1,NLRP10,PAK1,PAX3,PHF17,PLAGL1,POGK,PTGS2,RGS2,SDPR,SH3KBP1,SMAD1,SOX4,SPP1 ,STAT6,THBD,TNFAIP2,TNFRSF10A,TNFRSF11B,TNFRSF21,TRAF3IP2

**Table 8:** Top transcription factors by Ingenuity Pathway Analysis in IR scenario.

Transcription Regulator	p-value	Target molecules in dataset
**TP53**	5.78E-05	ADRB2,APAF1,BTG2,CDKN1A,DDIT4,DGKA,FAS,GDF15,MDM2,MYBL1,MYC,NOTCH1,PHLDA3,TAP1,TNFRSF10A
**ESR2**	9.03E-05	CDKN1A,CXCL12,EREG,MYC
**NFkB (complex)**	1.10E-04	CCNB2,CDKN1A,CXCL10,CXCL12,FAS,GDF15,MDM2,MYC,TAP1
**TP63**	1.19E-04	APAF1,CDKN1A,FAS,MDM2,TNFRSF10A
**JUN**	1.43E-04	CDKN1A,CXCL10,DUSP1,EREG,JUN,MYC

### Validation

**Figure 9:**
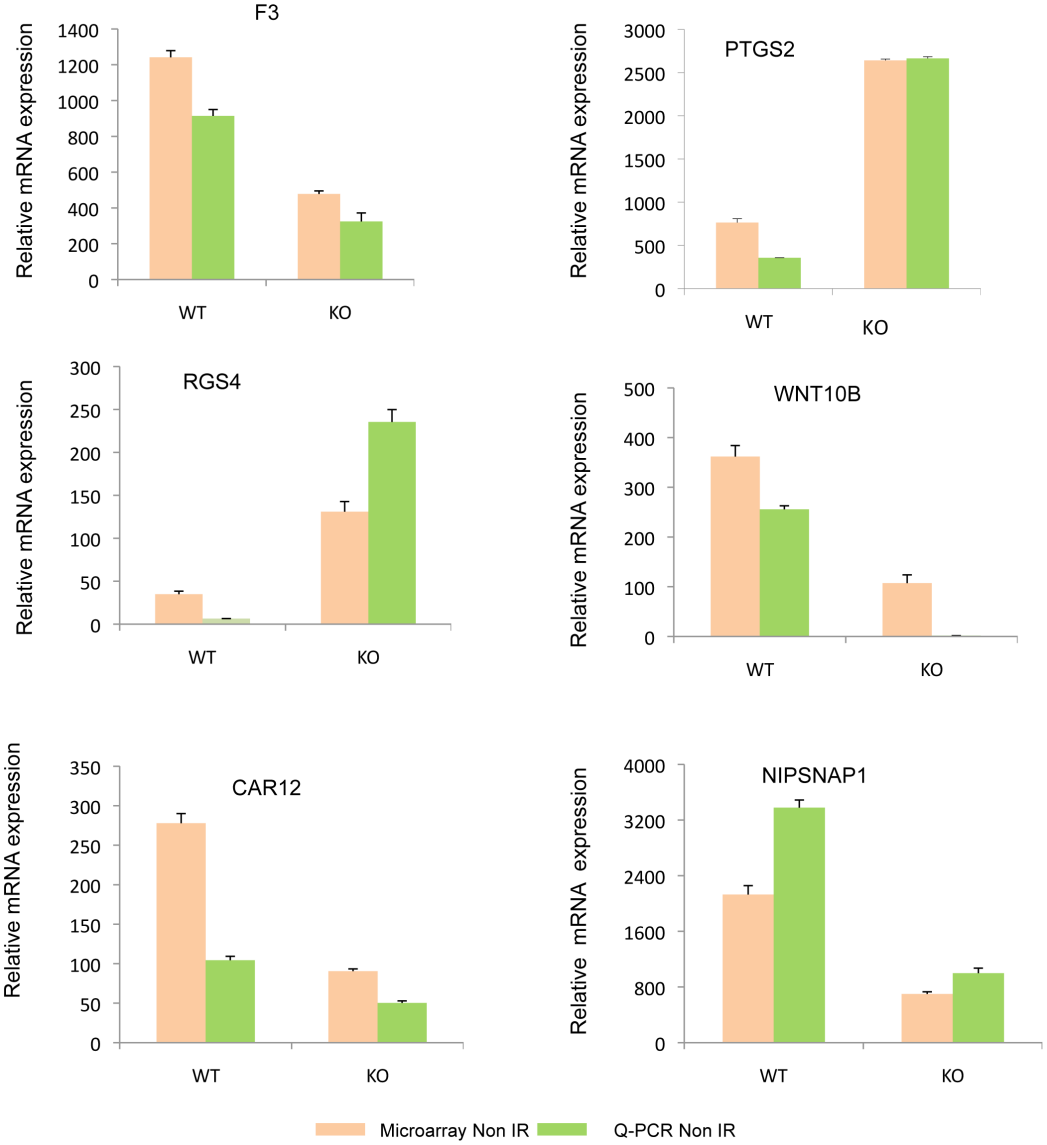
Microarray validation of targets regulated by PAK1. The RT-qPCR was performed in duplicates for each of the triplicate WT and *Pak1-*KO RNA sample from MEFs. The relative mRNA expression levels is represented in the figure and the p-values were computed. The orange bars show the expression data from Microarray and the green bars represent expression levels obtained by q-PCR. The Ct values of the genes were normalized with the Ct values of 18S.

**Figure 10:**
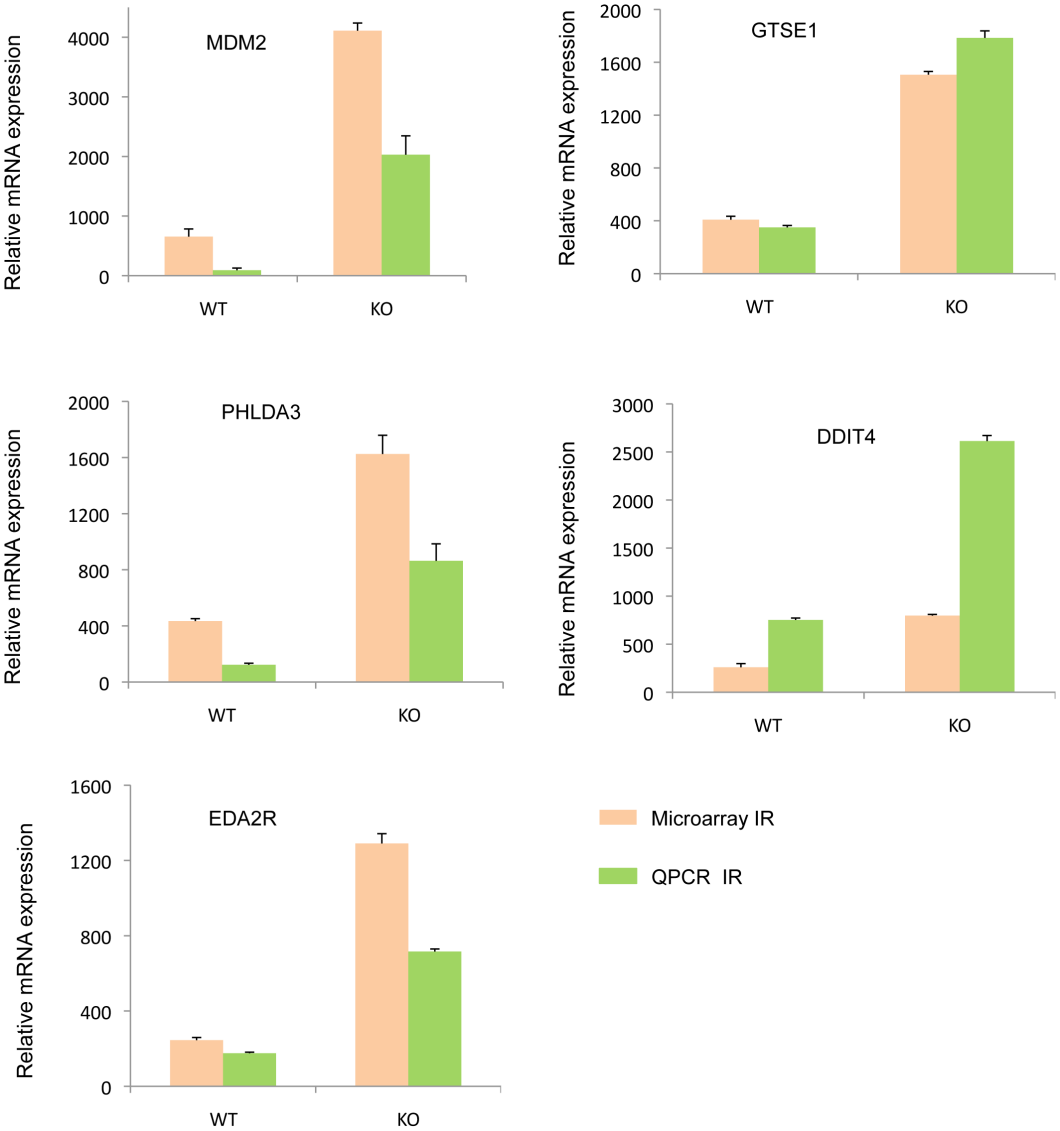
Microarray validation of targets regulated by PAK1 and responsive to IR. The RT-qPCR was performed in duplicates for each of the triplicate WTIR and KOIR RNA sample. The WT and *Pak1*-KO RNA samples from MEFs were subjected to ionizing radiation. The relative mRNA expression is represented in the figure and the p-values were computed. The orange bars show the expression data from Microarray whereas the green bars represent expression levels obtained by q-PCR. The Ct values of the genes were normalized with the Ct values of 18S.

To validate the results obtained from microarray analysis we chose several differentially expressed genes that are regulated by Pak1. The selection of the genes for validation was based on the interest of laboratory, role of the gene in top functions and pathways and significant fold change regulation. They include *Ptgs2* (3.3 fold upregualted), *Tmsb4x* (3.2 fold upregulated), *Wnt10b* (2.8 fold down regulated), *F3* (2.5 fold down regualted), *Rgs4* (upregualted by 3.4 fold change), *Nipsnap1* (3 fold down regulated), and *Car12* (2.9 down regulated) from the non–IR scenario. (Figure 9) and *Ddit4* (14 fold upregulation), *Eda2r* (5 fold change upregualted), *Gtse1* (3.5 fold upregualted), *Mdm2* (6 fold upregualted), *Phlda3* (3.8 fold upregulated), *Pmaip1* (9 fold upregualted), from the IR scenario (Figure 10) *DDdit4*, *Phlda3*, *Pmaip1* and *Eda2r* were all highly upregulated due to loss of Pak1 and were predicted to be involved in cell death due to ionizing radiation. Gtse1 and Mdm2 were important targets in P53 signaling which was the top pathway in the IR scenario. The protein encoded by *Gtse1* is only expressed in the S and G2 phases of the cell cycle. During this phase it colocalizes with cytoplasmic tubulin and microtubules. In response to DNA damage, the protein aggregates in the nucleus and binds P53, thus pushing it out of the nucleus and suppressing its ability to induce apoptosis. [40]. This protein shares many functions as PAK1 like the association with cytoplasm dynamics and its localization in the nucleus. Such correlations bring out interesting association which one might want to explore. From the non-IR dataset, we chose to validate the alteration of Thymosin beta 4, X-linked (*Tmsb4x*) and regulator of G-protein signaling 4 (*Rgs4*) that were up-regulated in the Pak1-KO MEFs as compared with WT MEFs revealed by microarray analysis. *Tmsb4x* gene encodes an actin sequestering protein which binds to and sequesters actin monomers (G actin) and therefore plays a role in regulation of actin polymerization [41], [42]. The protein is also involved in cell proliferation, migration, and differentiation. Recent study revealed that *Tmsb4x* is likely to be ERβ target gene identified by microarray analysis of altered gene expression in ERβ-overexpressing HEK293 cells [43], and induced under hypoxic conditions in murine melanoma B16 (F10) cells [44]. Overexpression of *Tmsb4x* is associated with increased invasion of SW480 colon carcinoma cells and the distant metastasis of human colorectal carcinoma [45]. Regulator of G protein signaling (RGS) proteins are GTPase-activating proteins for heterotrimeric G proteins. RGS4, has been to increase cell adhesion and migration in human glioma cells [46] and promote cell survival of thyroid cancer cell [47]. By contrast, the tumor suppressor function of RGS4 was also proposed. Also RGS4, is associated with cancer cell motility and shown to selectively inhibit *Rac*-activated lamellipodia formation in breast cancer cells [48]. Pak1 is also known to induce lamellipodia, filopodia and membrane-ruffle formation [1], [3]. More importantly, both proteins have been shown to play a critical role in breast cancer manipulating cell motility but no connection between them is reported so far. Further studies are needed to uncover such relationships between molecules which might lead to novel therapeutic interventions. Till now no studies have reported the regulation of these genes by Pak1. Based on these observations, we hypothesize that these targets are novel and their regulation by Pak1 could be further explored.

## Conclusions

In summary, in this study we provide the first genome-wide analysis of Pak1 target genes upon exposure to a DNA damaging agent. A total of 732 probe sets were identified to be differentially expressed in wild-type and Pak1-knockout cells and 199 that were regulated by Pak1 and were responsive to ionizing radiation. Gene ontology, KEGG pathway and IPA network analyses revealed that these differentially expressed genes are involved in specific cellular processes, signaling pathways, and gene networks. The genes that were regulated by Pak1 in IR scenario were specifically involved in cell cycle arrest and apoptotic events in response to DNA damage. This is important because the inability to control cell cycle progression is the root cause of cancer development. To target one of the most elementary steps is very important in order to cure a disease. Genes involved in cellular growth proliferation, DNA replication, recombination and repair formed the most significant cluster in our dataset which leads us to the hypothesis that most of the genes affected by Pak1 knock out are involved in repairing the DNA damaged by ionizing radiation which lead to better cell survival. This data set opens new avenues of understanding the genomic regulation of these genes by Pak1 which can provide an insight into genomic instability of the cancer genome. The data analysis also revealed a genomic regulation between Pak1 and major transcriptional regulators such as P53, NF-kB, JUN that control a big cluster of targets. Genome wide microarray analysis has been performed for the first time to indentify the role of Pak1 and its regulatory targets during genotoxic insults such as DNA damage. In contrast, in the non-IR condition where functions of Pak1 in areas such as developmental process and metabolic process functions still remain very poorly defined, our data provides a rich source for future investigation. The data analysis also reveals class of signaling molecules, histones and enzymes being significantly affected by the loss of Pak1. The association of Pak1 with histones and transcription factors provides new targets that can be tested for its association with nucleus and the chromatin. Accumulating evidence has pointed out that there are several potential mechanisms responsible for PAK1 regulation of gene transcription. First, nuclear PAK1 associates with chromatin and regulates histone modifications such as phosphorylation of histone H3 at serine 10, which in turn directs gene transcription [13], [14]; second, PAK1 may interact with and phosphorylate transcription factors, transcription coregulators, or chromatin remodeling factors, thus regulating the bindings of the transcription machinery to nucleosomal DNA and consequently, gene transcription. A case in point is that PAK1 interacts with and phosphorylates transcriptional corepressor SHARP and enhances SHARP-mediated repression in Notch signaling [49]. Similarly the families of signaling molecules and enzymes can be tested for various developmental and metabolic processes. The presented data on one hand re-emphasizes the overall known function of Pak1 and the identification of new targets also definitely points to the unexplored biological functions of Pak1. Collectively, these identified candidate targets of Pak1 and their associated functional analysis may provide new avenues to further explore the possibility of developing these targets as biomarkers as well as identifying novel functions and regulatory mechanisms of Pak1.

## Materials and Methods

### Cell culture, Ionizing radiation treatment and RNA isolation

Pak1 WT and Pak1 KO MEFs [50] were maintained in DMEM/F-12 medium supplemented with 10% fetal bovine serum and 1× antibiotic-antimycotic solution in a humidified 5% CO_2_ at 37°C. All cell culture reagents were purchased from Invitrogen (Carlsbad, CA). Cells were irradiated with a Nasatron 137Cs irradiator (U.S. Nuclear, Burbank, CA) at a dose rate of 3.04 Gy/min at room temperature. Control cultures were identically processed but not irradiated. Total RNA was isolated using TRIzol reagent (Invitrogen, Carlsbad, CA) following the protocol provided by the manufacturers, and then purified using RNeasy Mini Kit (Qiagen, Valencia, CA) and tested for integrity on RNA 6000 NanoChips using an Agilent 2100 Bioanalyzer (Agilent Technologies, Santa Clara, CA).

### Microarray gene expression assays and data analysis

Microarray gene expression assays and data analysis has been performed as described previously [51]. In brief, rRNA reduction of the purified total RNA samples was performed using the RiboMinus™ Transcriptome Isolation Kit (Invitrogen, Carlsbad, CA). The rRNA-reduced samples were then amplified and labeled using the GeneChip® Whole Transcript cDNA Synthesis/Amplification Kit and GeneChip Whole Transcript Terminal Labeling Kit (Affymetrix, Santa Clara, CA). Labeled single-stranded DNA samples were then prepared in a volume of 220 µl of hybridization mixture using the Hybridization Module of the GeneChip Hybridization, Wash and Stain Kit from Affymetrix. For each sample, 200 µl of hybridization mixture was hybridized on a GeneChip Mouse Exon 1.0 ST Array (Affymetrix, Santa Clara, CA), which contains more than 266,000 probe sets, at 45°C with 60 rpm rotation in an Affymetrix Hybridization Oven 640 for 17 h. Following hybridization the arrays were scanned on an Affymetrix GeneChip Scanner 3000 7G, after the wash and staining on an Affymetrix Fluidics Station 450.

For microarray data analysis, results were saved as a .CEL file using the Command Console for each of the arrays. Data were processed in GeneSpring GX by statistical analysis using unpaired *t* test with *p* value computations done asymptotically and multiple corrections as Benjamin Hochberg, FDR were applied with a *p* value cut-off of 0.05. Heat map analysis of the identified genes for individual arrays was performed using MultiExperiment Viewer version 4.4. Gene ontology (GO) analysis was performed using GeneSpring GX 10.0.2. The data discussed in this publication has been deposited in NCBI's Gene Expression Omnibus [52] and are accessible through GEO Series accession number GSE47503 (http://www.ncbi.nlm.nih.gov/geo/query/acc.cgi?acc=GSE47503).

### Pathway and functional analysis

Gene Ontology analysis from GeneSpring GX 10.0.2. was used to identify GO terms , molecular functions and biological processes at a p value cut off set to 0.1. The datasets of differentially expressed genes in the WT and Pak1-KO as well as PAK1 regulated IR responsive genes, were analyzed using Ingenuity Pathway Analysis to discover relationships between the genes. DAVID 6.7 was used to perform the pathway analysis using the KEGG pathways. The Fisher's exact test was used to identify significant functions and pathways represented within the respective datasets.

### RT-qPCR and Western blot analysis

RT-qPCR and Western blot analysis were performed following the protocol described previously [53], [54]. In brief, total RNA was isolated by using TRIzol reagent (Invitrogen), and 2 µg of total RNA was reverse-transcribed using the SuperScript III First-strand synthesis system for reverse transcriptase-PCR (Invitrogen). Quantitative PCR (QPCR) was done by using iQ™ SYBR Green Supermix (Bio-Rad Laboratories, Hercules, CA) on an iCycler iQ real-time PCR detection system (Bio-Rad Laboratories, Hercules, CA). The real time PCR Ct values for specific genes were normalized to housekeeping control 18S. Primer sequences are available on request.

For Western blot analysis of Pak1 expression in the WT and Pak1-KO MEFs, protein extracts were prepared by lysing the cells in the RIPA lysis buffer containing 50 mM Tris-HCl (pH 7.4), 1% Nonidet P-40, 150 mM NaCl, 1 mM ethylenediaminetetraacetic acid, 0.25% sodium deoxycholate, 1×protease inhibitor mixture (Roche, Indianapolis, IN), and 1× phosphatase inhibitor mixture I and II (Sigma, St. Louis, MO), and protein concentrations were determined by using Bio-Rad *DC* Protein Assay reagents (Bio-Rad Laboratories, Hercules, CA). Cell lystaes were then resolved by sodium docecyl sulfate-polyacrylamide gel electrophoresis, transferred to nitrocellulose membranes, and incubated with respective antibodies. Rabbit polyclonal anti-Pak1, mouse monoclonal anti-vinculin primary antibodies, and horseradish peroxidase-coupled secondary antibodies were obtained from Bethyl Laboratories (Montgomery, TX), Sigma-Aldrich (St. Louis, MO), and Amersham Biosciences (Piscataway, NJ), respectively.

## Supporting Information

Table S1 **Pak1 target genes with a fold change ≥±2.0 and with the p-value<0.05.**
(XLSX)

Table S2 ***Pak1*** **influenced genes after ionization radiation treatment with a fold change ≥±2.0 and with the p-value<0.05.**
(XLSX)

Table S3 **Gene ontology terms involved in the WT vs PAK1 KO samples.**
(XLSX)

Table S4 **Gene Ontology terms for** ***Pak1*** **influenced DNA damage response genes.**
(XLSX)

Table S5 **KEGG pathways by DAVID tool in non-IR scenario.**
(XLSX)

Table S6 **KEGG pathways by DAVID tool in the IR scenario.**
(XLSX)

Table S7 **The top networks by IPA in both non-IR and IR scenarios.**
(XLSX)

Table S8 **Over or under represented gene families in Pak1 target genes and** ***Pak1*** **influenced genes after ionization radiation.**
(XLSX)

Table S9 **Transcription factors regulating Pak1 targets.**
(XLS)

Table S10 **Transcription factors regulating Pak1 influenced DNA damage response genes.**
(XLS)
